# Changes in Physical Activity Are Associated with Corresponding Changes in Psychological Well-Being: A Pandemic Case Study

**DOI:** 10.3390/ijerph182010680

**Published:** 2021-10-12

**Authors:** Micael Dahlen, Helge Thorbjørnsen, Hallgeir Sjåstad, Petra von Heideken Wågert, Charlotta Hellström, Birgitta Kerstis, Daniel Lindberg, Jonas Stier, Maria Elvén

**Affiliations:** 1 Stockholm School of Economics, SE-11383 Stockholm, Sweden; micael.dahlen@hhs.se; 2 Centre for Applied Research (SNF), Norwegian School of Economics, 5045 Bergen, Norway; 3 Department of Strategy and Management, Norwegian School of Economics, 5045 Bergen, Norway; hallgeir.sjastad@nhh.no; 4 Division of Physiotherapy, School of Health, Care and Social Welfare, Mälardalen University, SE-72123 Västerås, Sweden; petra.heideken.wagert@mdh.se (P.v.H.W.); maria.elven@mdh.se (M.E.); 5 Division of Public Health Sciences, School of Health, Care and Social Welfare, Mälardalen University, SE-72123 Västerås, Sweden; charlotta.hellstrom@mdh.se; 6 Division of Caring Sciences, School of Health, Care and Social Welfare, Mälardalen University, SE-72123 Västerås, Sweden; birgitta.kerstis@mdh.se; 7 Division of Social Work, School of Health, Care and Social Welfare, Mälardalen University, SE-72123 Västerås, Sweden; daniel.lindberg@mdh.se (D.L.); jonas.stier@mdh.se (J.S.)

**Keywords:** happiness, life satisfaction, well-being, physical activity, exercise, inactivity, COVID-19

## Abstract

Societal crises and personal challenges are often followed by substantial changes in physical activity. Is there a link between such changes and psychological well-being? Seeking to answer this question, we conducted a correlational study on a representative sample in Sweden during the first year of the COVID-19 pandemic (N = 1035). About 49% of the sample had decreased their physical activity compared to their self-reported activity level prior to the pandemic, whereas 32% had increased it. The results showed a positive and robust association between changes in daily activity level and corresponding changes in psychological well-being. Specifically, individuals who had reduced their physical activity over the last year reported lower life satisfaction than before, and individuals who had increased their physical activity reported higher life satisfaction than before. The amount of complete physical inactivity (sitting) showed a similar pattern as the exercise data, meaning that individuals who reported increasing inactivity per day also reported a greater decline in life satisfaction. Additional analyses showed that the association between daily activity level and life satisfaction was somewhat stronger for men than for women, but there was no difference when comparing individual versus organized activities. The current study was based on a cross-sectional design, measuring self-reported change over time. Recent work from other research teams have used longitudinal data and experience-sampling in different settings, finding similar results. We conclude that there is good reason to recommend physical exercise as a coping strategy in difficult times.

## 1. Introduction

In this research paper, we ask the following question: Is there a mutual connection between changes in physical activity and psychological well-being? If so, in what ways are the two variables connected, for what type of physical activities, and for whom? To address these questions empirically, we report the results from a large correlational study from Sweden during the COVID-19 global pandemic, consisting of self-reported measures comparing past and present life conditions among 1035 participants from a nationally representative sample.

As we write this paper (August 2021), the novel coronavirus disease has been spreading across the world for more than a year, resulting in more than 200 million infections and 4 million deaths according to the World Health Organization [1]. Until effective vaccines can be widely distributed, governments all over the world have implemented a wide array of strict policy measures to reduce the further spread of the virus, such as social and physical distancing, and temporary “lockdown” interventions in society. In addition to slowing down the coronavirus to save human lives, pandemic restrictions have also led to a substantial decline in physical mobility worldwide. Indeed, routines and habits involving physical activity have changed quickly, and at large scale.

For example, a global study of about half a million people in 187 countries found that the average number of steps per day decreased across all countries, averaging a reduction of 27% (and ranging from −7% in Sweden to −49% in Italy), within the first month of the pandemic [2]. A study using Google mobility across 42 nations found a reduction of 35% in physical movement patterns during the first wave of the pandemic [3], and physical activity data released by [4] show a similar pattern. In response, many scholars and governments across the world have recognized the dangers of reduced physical activity to people’s physical and mental health and recommend maintaining or even increasing one’s outdoor activity level to cope with the pandemic [5,6,7].

This makes it a widespread assumption that physical activity is generally an important predictor of psychological well-being, and that long-lasting *activity change* may be particularly important during the challenging times of a pandemic. Some studies find that the decrease in physical activity may differ between demographic cohorts [8] and types of activity [9], where some groups have even increased their levels of physical activity during the pandemic [10,11]. This presents both a research opportunity and a societal urgency to examine how real-life changes in physical activity have been shaping peoples’ psychological well-being, and whether such an effect may differ depending on the direction of change (increase or decrease), type of physical activity, and demographic variables such as age and gender.

While there are some recent studies investigating the relationship between changes in physical activity and the present state of psychological well-being during the pandemic [9,10], we will focus on the possible connection between *changes* in physical activity to *changes* in well-being since the outbreak of COVID-19. In this paper, we report a study of a nationally representative sample of 1035 Swedes, which tests the general hypothesis that a relative *increase* in physical activity over time is associated with a corresponding *increase* in psychological well-being, based on self-reported change from before vs during the pandemic. In addition, we also compare the nature of this association for men and women, and between different types of physical activity (organized vs. individual).

## 2. Physical Activity and Well-Being during the Pandemic

The positive relationship between physical activity and psychological well-being is well documented in the research literature. For example, one study on children found that those who engaged in physical activity at school [12] and outside of school [13] were happier than those who did not. Another study found that more physically active elderly people tend to live longer and happier lives than those who are less active [14]. More generally, Zhang and Chen [15] reported a systematic review of 23 studies of the relationship between physical activity and happiness, which found a consistent and significant positive relationship across age and geography. Drawing on a combination of cross-sectional, longitudinal, and experimental studies, the authors concluded that as little as 10 min of physical activity per week or one day of exercise per week might result in increased levels of happiness. In terms of underlying mechanisms, mediation analyses suggested that the positive effect of physical health on happiness is partly driven by health effects and better social functioning. Similar results have been found for the positive link between physical activity and life satisfaction [16], which is a core component of psychological well-being in addition to “happiness”, meaning the balance between positive vs. negative emotions [17].

When it comes to the internal relationship between physical activity and well-being, a review by Veenhoven [18] concluded that bi-directional effects are likely present. In other words, physical activity might make people happier, but happy people might also be more likely to engage in physical activity. This notion makes the interpretation of the recent studies on physical activity and well-being during the pandemic particularly tricky, as they are based on correlations between changes in physical activity with the present state-measures of well-being. In addition to replicating this basic pattern, the current investigation is focused on the connection between self-reported *change* in physical activity and *change* in psychological well-being over time, and to what extent these changes are internally correlated in the predicted direction.

### 2.1. Hypothesis 1

We now turn to our specific hypotheses in the current study. First (H1), we predict that self-reported change in physical activity during the COVID-19 pandemic is positively correlated with self-reported change in psychological well-being. Specifically, people who are being *more* physically active than before will also tend to have become more satisfied with their lives than those who report similar or *less* physical activity than before.

The predicted pattern would fit well with the finding from Tkach and Lyobmiursky’s [19] study on 500 ethnically diverse undergraduates, suggesting that exercise is one of the most common (and seemingly most impactful) happiness-promoting strategies there is. It would also resonate well with reviews on the general association between physical activity and happiness [15,18], described above, and with a recent study of daily emotional well-being during the pandemic [20]. Using experience-sampling methods, the latter study collected around 3000 daily episodes among 200 participants and found that physical exercise and other outdoor activities was the strongest predictor of positive affect (and was negatively correlated with negative affect). At the other end of the spectrum, they found that updating oneself about the current COVID-19 situation from the news had the weakest association with positive affect and the strongest association with negative affect.

We also predict that a *decrease* in physical activity will be associated with *reduced* psychological well-being during the pandemic. This prediction extends previous research finding that daily episodes which are associated more with negative affect than positive affect did not include physical activity [20], such as doing nothing, working, or studying, watching TV, or using social media. To summarize, our first and primary hypothesis (H1) predicts that positive change in physical activity will be associated with a corresponding positive change in psychological well-being, and conversely, that negative change in physical activity will be associated with a negative change psychological well-being.

### 2.2. Hypothesis 2

As our second hypothesis (H2), we predict that self-reported change in physical activity will have greater impact on men’s than women’s well-being. We form this expectation based on previous findings that, on overage, men are usually more oriented towards physical activity than women, which could suggest that a negative change in physical activity would be more preference-inconsistent and consequential for men. For example, a recent study of almost two million people in 168 countries found that insufficient physical activity (defined as less than either 150 min of moderate intensity or 75 min of vigorous intensity per week) is significantly less prevalent among men (average 23 percent) than women (32 percent) [21]. Indeed, a U.S. study found that men reported much higher rates of regular and sustained physical activity than women [22].

Research on school children and adolescents has found a similar pattern, in which boys are more physically active than girls, and seem to be more motivated and confident to engage in physical activity and value it more in life [23,24]. Other studies suggest that this gender gap in physical activity continues into adulthood [25]. In investigating amateur and recreational athletes’ physical exercise during COVID-19, Lautenbach et al. [26] found that female athletes were less motivated to train in comparison to male athletes.

Seen as a whole, we predict that men may, on average, be more likely to use physical activity as a coping strategy during the pandemic and will also derive greater well-being from it than women. Conversely, we predict that men will respond more negatively to reductions in physical activity. In summary, the central prediction in our second hypothesis (H2) is that self-reported change in physical activity will be more strongly associated with self-reported changes in psychological well-being for men than for women.

### 2.3. Hypothesis 3

Previous research on physical activity during the pandemic studied effects across different types of activity, such as exercising with others or alone [5], location [8], indoor or outdoor [27], and vigorous, moderate, or walking intensity [9]. In the present study, we differentiate between organized and individual forms of physical activity.

Since organized and individual forms of activity may differ both in the degree of pandemic regulation (i.e., more restrictive for organized exercise) and individual control and agency (i.e., larger for individual exercise), our third hypothesis (H3) was the prediction that the association between self-reported change in physical activity and psychological well-being would differ depending on the *type* of activity. Due to the social nature of organized exercise, it could be the case that a decline in that type of activity would be more strongly related to reduced well-being than a similar decline in individual activity (H3a). Conversely, it could also be the case that the agentic and autonomous aspect of individual activity would have a stronger relationship with self-reported change in well-being (H3b), which provided the basis for a bi-directional Hypothesis 3.

## 3. Method

The current research was based on a cross-sectional study design, relying on self-reported change in life conditions over time. A demographically representative sample of 1035 Swedes were recruited from the Novus Sweden panel during the first two weeks of December 2020 (49.5% females, mean age = 50.6 years, age span = 18–79 years; response rate = 52%), during the second wave of the COVID-19 pandemic. The participants anonymously filled out an online survey about their physical activity level and psychological well-being before and during the pandemic. The study was conducted in accordance with the Declaration of Helsinki, and all participants provided their informed consent to take part in this research. All personal data connections were deleted after the material was collected and were not accessible to the researchers in the present study.

### Measures

Change in physical activity was measured with the International Physical Activity Questionnaire Short form (IPAQ-SF) from Craig et al. [28], asking respondents to report approximately how many minutes they engaged in (a) vigorous activity, for example, aerobics and running, (b) moderate activity, for example, cycling and gardening, and (c) walking. For each measure, respondents first reported the number of minutes “in a normal week, 1 year ago (before the pandemic)” and then reported the number of minutes in “the past 7 days”. The three categories were added together for two counts of total physical activity level (1 year ago, and in the past 7 days). We then subtracted the first count (physical activity 1 year ago) from the second count (physical activity in the past 7 days) for our primary predictor variable, average change in physical activity level over time.

Similar to previous studies, we also included two measures (“number of minutes in a normal week, 1 year ago”, and number of minutes in the past 7 days”) of sedentary behavior (sitting) and calculated the average change over time using the same procedure as described above.

Changes in organized and individual physical activity was measured by asking respondents to rate how much they had increased or decreased these activities during the pandemic on a scale ranging from −3 (decreased significantly) to +3 (increased significantly).

Change in psychological well-being was measured with two global items of life satisfaction: “How satisfied are you with life right now, compared to 1 year ago (before the pandemic)?” and “How would you rate your well-being right now, compared to 1 year ago (before the pandemic)?” on an 11-point scale ranging from −5 (much less than before) to +5 (much more than before). The two items were averaged into an index of self-reported change in psychological well-being (Cronbach’s alpha = 0.81; *r* = 0.69, *p* < 0.001).

To replicate previous research findings, we also included a similar two-item global measure of current psychological well-being, which is commonly used in well-being research [17].: “How satisfied are you with life right now?” and “How would you rate your well-being right now?” on an 11-point scale from 0–10 (Cronbach’s alpha = 0.91; *r* = 0.83, *p* < 0.001).

## 4. Results

As can been seen from Table 1, 32.2% of the sample reported higher physical activity than prior to the pandemic, where 49.3% of the participants had decreased their physical activity and 18.6% reported no change at all. Thus, in a nationally representative sample of Swedes (N = 1035), there was substantial variation in self-reported behavioral responses to the pandemic in the terms of physical activity level. Scientifically speaking, this provides a good test case to examine corresponding differences in psychological well-being over the same time period. There was no significant gender difference in physical activity change (Chi-square = 1.39, *p* = 0.50).

H1. In support of our first and primary hypothesis (H1), the mean change in general life satisfaction was positive (0.22) in the group who reported an increase in physical activity over the last year, and negative (−0.33) in the group who reported a decrease in their physical activity level. This difference was robust and statistically significant, *F* = 6.76, *p* < 0.001. Providing similar results, a Chi-square test showed that positive changes in life satisfaction were significantly more common in the group that had increased their physical activity level, whereas negative changes in life satisfaction were significantly more common in the decreased activity group (*X*^2^ = 18.66, *p* = 0.006).

For comparison, we also included current life satisfaction in a simple correlation analysis, to provide a broader outcome measure. This analysis was based on the full continuous scale measures for all three variables. As can be seen from Table 2, the results showed a positive and significant correlation between change in physical activity and current life satisfaction (*r* = 0.07, *p* = 0.017), whereas the strongest correlation was found with our primary outcome measure, namely change in life satisfaction over time (*r* = 0.12, *p* < 0.001). Thus, consistent with our first hypothesis, a positive change in physical activity was associated with a corresponding positive change in psychological well-being, and conversely, a negative change in physical activity was associated with a negative change psychological well-being.

To explore these results further, we ran a linear regression with change in total physical activity (*Standardized Beta* = 0.11, *t* = 3.46, *p* < 0.001) and change in sedentary (sitting) behavior (*Standardized Beta* = −0.16, *t* = −5.23, *p* < 0.001) as independent variables, finding that increasing physical activity and decreasing sedentary behavior were associated with positive changes in life satisfaction, whereas decreasing physical activity and increasing sedentary behavior were associated with negative changes in life satisfaction (*R*^2^ = 0.047, *F* = 25.14, *p* < 0.001).

As an exploratory analysis, we compared the effects of total level of physical activity in past and present with relative *change* between these time points in physical activity. This analysis found that change in physical activity from one year ago to present was a positive and significant predictor of change in psychological well-being (*Standardized Beta* = 0.14, *t* = 4.52, *p* < 0. 001), although physical activity 1 year ago (*Standardized Beta* = 0.00) and physical activity in the past 7 days (*Standardized Beta* = 0.03, *t* = 0.94, *p* = 0.35) were *not* significant predictors when treated in isolation. Thus, it appears to be the case that changes in physical activity have played a greater role in psychological well-being during the pandemic than the absolute activity level.

H2. Turning to our second hypothesis (H2) on the role of gender, the bivariate correlation between change in physical activity and change in life satisfaction was stronger for men (*r* = 0.19) than for women (*r* = 0.09), *z* = 1.64, *p* = 0.05. Although the strength of this finding was modest, it may suggest that physical activity has played a greater role for the psychological well-being of men than women during the pandemic. Hence, H2 received not strong, but marginal support.

H3. Table 1 lists the sample percentages that increased, decreased or did not change their levels of *organized* and *individual* physical activity during the pandemic. Organized physical activity decreased significantly more than individual activity (55.1% vs. 36.9%), and individual physical activity increased significantly more than organized activity (26.9% vs. 9.4%), *X*^2^ = 380.1, *p* < 0.001), perhaps as you would expect in a pandemic. An additional analysis of these exercise patterns showed that 26.8% of those who decreased their amount of organized physical activity instead increased their individual physical activity, *X*^2^ = 380.8, *p* < 0.001, suggesting the emergence of compensation behavior.

Turning to the specific test of our third and final hypothesis, consisting of a bi-directional prediction of whether organized (H3a) or individual (H3b) physical activity would play the greatest role for psychological well-being, there was no significant difference in the correlations between change in organized vs. individual physical activity on corresponding changes in life satisfaction. Hence, H3 was not supported.

## 5. Discussion

The current paper investigated whether changes in physical activity are connected to corresponding changes in psychological well-being over time. Based on survey data from the COVID pandemic and more than 1000 participants from a representative sample in Sweden, our findings suggest that the answer is ‘yes’. At least in the current setting, individuals who reduced their physical activity reported *lower* life satisfaction than one year earlier, and conversely, individuals who had increased their physical activity reported *higher* life satisfaction than before. Thus, we found clear support for the primary hypothesis in the current study (H1). This finding resonates well with previous research suggesting that exercise is an effective happiness-promoting strategy [19], and that level of physical activity has been positively linked to happiness [15,18] and daily emotional well-being during the pandemic [20]. Our findings also echo previous research associating inactivity with more negative emotions [20]. Physical activity might not only be a matter of life and death during the pandemic, in the sense that better physical fitness is associated with lower mortality [29,30], but also in reducing death anxiety and consequently increasing well-being [31].

We did not find any difference for organized versus individual forms of physical exercise (H3 was not supported), but the strength of the general association with psychological well-being was somewhat stronger for men than for women (H2 was supported). This may suggest that physical exercise is a particularly important coping strategy for men during global pandemics and other crises requiring major adjustments. For both women and men, relative change in physical activity seemed to play a greater role in psychological well-being than absolute levels. As such, the current study provides clear evidence for the importance of *behavior change* over time for corresponding changes in well-being.

Specifically, the present study adds to our understanding of how changes in physical activity are associated with well-being by gauging *change* in well-being, rather than the more frequently used measure of present (absolute level) well-being. This resonates with the notion that happiness is to a large extent transitory [32], so that static measures may underestimate the impact of dynamic events [33]. Indeed, while the present study found a significant correlation between change in physical activity with present well-being similar to previous studies, the association was stronger with change in well-being. It may not be entirely easy for people to assess how much their well-being has changed, but on the other hand, it rids them from the difficulty of disregarding situational factors, what Lykken and Tellegen [34] call stochastics in their classic paper, that unknowingly impact happiness and well-being at any given moment [35].

In summary, replicating and extending previous research on the connection between physical activity and well-being in everyday life [20], the current study shows that changes in physical activity are positively associated with changes in psychological well-being during the COVID pandemic. Although the current research was exclusively based on a cross-sectional design with only a one-shot measure of self-reported change, the findings are consistent with recent evidence from a longitudinal study in Germany [36] which found that large reductions in physical activity were a leading risk factor for developing clinical depression during the pandemic.

## 6. Conclusions

Seen as a whole, the available research provides converging evidence across multiple methods for a positive relationship between physical activity and psychological well-being over time. In our view, this suggests that there is good reason both for scientists and policy-makers to extend their recommendation of physical activity, exercise, and outdoor activities as preventive medicine in difficult times.

Seen in isolation, an important limitation with the current study is that it was conducted in a single cultural setting, Sweden, using purely observational methods. Governmental responses to the pandemic varied substantially across countries and cultures. Initially, Sweden decided to not implement very strict measures in the form of societal lockdown, which left more individual and collective responsibility to its citizens. It should therefore be examined whether the current results generalize to different countries that implemented different policies during the first wave of the pandemic. In terms of research design, additional evidence from multiple methods and a broader collection of cultural samples are needed to make causal inference and support more generalizable conclusions. Ideally, future studies should apply time-series (panel) data in combination with experimental study designs, rather than relying on self-reported change alone. Additionally, physical mobility data and the longitudinal development pattern of such data during the pandemic would certainly complement the self-reported cross-sectional data in the current research.

However, when considering the current findings in the context of the wider research literature, it appears to be rather clear that there *is* a positive and robust relationship between physical activity and corresponding changes in psychological well-being. In our view, a plausible interpretation of this pattern is that the causal pathway between these variables is bi-directional, as suggested by Veenhoven [18]: Changes in physical activity is followed by subsequent changes in psychological well-being, but changes in psychological well-being might also initiate similar changes in physical activity. In terms of mechanisms, a systematic review by Zhang and Chen [15] suggested that health benefits and better social functioning are important mediators behind the happiness effect of physical activity. Reclaiming a sense of mastery and control in a difficult life situation, perhaps in combination with future optimism, might be another mechanism in the ongoing dynamic between acting better and feeling better. The precise nature of this relationship remains a topic for future work in social science, medicine, and behavioral health research.

Finally, it would be interesting to expand the scope and learn more about other health-specific factors in determining physical activity and psychological well-being over time, such as the role of weight and obesity. The current study did not collect any information about this factor. If overweight individuals are more at risk for developing severe health problems following coronavirus infection, it would be especially important to examine effective strategies that may promote the physical activity level in this group during global pandemics and other health crises in society.

## Figures and Tables

**Table 1:** Self-reported change in physical activity (PA) and psychological well-being, comparing before versus during the first year of the COVID-19 pandemic (N = 1035, Sweden). Highlighted in bold, the three columns show the mean change in psychological well-being for participants who either increased, did not change, or decreased their physical activity level over the last year.

	Increased PA	No Change in PA	Decreased PA
Percentage of sample (female/male)	32.2% (33.6%/30.8%)	18.6% (17.4%/19.7%)	49.3% (49%/49.5%)
Mean change in physical activity minutes/week	330.4	0	−531.8
Mean change in psychological well-being	**0.22 ^a^**	**−0.04 ^a,b^**	**−0.33 ^b^**
High intensity activity, mean change in minutes	47.7	−1.4	−143.8
Moderate activity, mean change in minutes	54.7	−2.1	−233.2
Walking activity, mean change in minutes	228.0	3.5	−154.9
Sedentary behavior (sitting), mean change in minutes	173.3	141.3	537.3
Organized PA change, percentage of sample	9.4%	35.4%	55.1%
Individual PA change, percentage of sample	26.9%	36.2%	36.9%

**Table 2:** Correlation matrix showing positive associations between self-reported change in physical activity, current psychological well-being, and change in psychological well-being over time during the first year of the COVID-19 pandemic (N = 1035, Sweden).

	Change in Well-Being	Current Well-Being
Change in well-being		
Current well-being	0.53 ***	
Change in physical activity	0.12 ***	0.07 *

*** *p* < 0.001, * *p* < 0.05.

## Data Availability

The data for this article is openly available at the OSF platform (DOI 10.17605/OSF.IO/W4GFE).
